# Remote liver ischemic preconditioning attenuates myocardial ischemia/reperfusion injury in streptozotocin-induced diabetic rats

**DOI:** 10.1038/s41598-021-81422-1

**Published:** 2021-01-21

**Authors:** Xinhao Liu, Hui Chen, Zhibing Yan, Lei Du, Dou Huang, Wei Dong Gao, Zhaoyang Hu

**Affiliations:** 1 Department of Anesthesiology, West China Hospital, Sichuan University, Chengdu, Sichuan China; 2 Laboratory of Anesthesiology and Critical Care Medicine, Translational Neuroscience Center, West China Hospital, Sichuan University, Chengdu, Sichuan China; 3 Department of Anesthesiology and Critical Care Medicine, Johns Hopkins University School of Medicine, Baltimore, MD 21287 USA

**Keywords:** Cardiovascular biology, Circulation, Cardiology, Diseases, Cardiovascular diseases

## Abstract

Diabetes mellitus (DM) exhibits a higher sensitivity to myocardial ischemia/reperfusion (I/R) injury and may compromise the effectiveness of cardioprotective interventions, including ischemic preconditioning. We previously found that liver ischemic preconditioning (RLIPC) could limit infarct size post I/R in non-diabetic rat hearts and further exerted anti-arrhythmic effects in diabetic or non-diabetic rats after myocardial I/R, however, little is known regarding the effect of RLIPC on infarct-sparing in diabetic hearts. In this study, we evaluated the protective effects of RLIPC on I/R injury in streptozotocin-induced type 1 diabetic rats. Type 1 diabetes mellitus was induced by one-time intraperitoneal injection of streptozotocin in Sprague–Dawley rats. Rats were exposed to 45 min of left anterior descend in (LAD) coronary artery occlusion, followed by 3 h of reperfusion. For liver ischemic preconditioning, four cycles of 5 min of liver I/R stimuli were performed before LAD occlusion. The cardioprotective effect of RLIPC was determined in diabetic rats. Compared to non-RLIPC treated DM rats, RLIPC treatment significantly reduced infarct size and cardiac tissue damage, inhibited apoptosis in diabetic hearts post I/R. RLIPC also improved cardiac functions including LVESP, LVEDP, dp/dtmax, and − dp/dtmax. In addition, RLIPC preserved cardiac morphology by reducing the pathological score post I/R in diabetic hearts. Finally, Westernblotting showed that RLIPC stimulated phosphorylation of ventricular GSK-3β and STAT-5, which are key components of RISK and SAFE signaling pathways. Our study showed that liver ischemic preconditioning retains strong cardioprotective properties in diabetic hearts against myocardial I/R injury via GSK-3β/STAT5 signaling pathway.

## Introduction

Cardiovascular disease is the most predominant cause of morbidity and mortality in patients with diabetes mellitus (DM). Abundant evidence has clearly demonstrated that patients with type 1 or type 2 diabetes are at high risk for ischemic heart disease and the mortality rate of acute myocardial infarction is dramatically increased in diabetic patients versus non-diabetic patients^1^. Myocardial ischemia reperfusion (I/R) injury is a significant complication of reperfusion therapy for myocardial infarction. Clinical and epidemiological studies indicate that diabetic hearts are more prone to I/R injury^2^, that is, diabetes is associated with larger infarcts and worse outcomes. Therefore, new strategies to limit infarction in clinical settings is of great importance.

Ischemic preconditioning, which is induced by episodes of controlled ischemia–reperfusion, was first demonstrated to have protective effect on myocardium against I/R injury in dog^3^. Later, it was shown that this type of myocardial protection against I/R injury can be induced by imposing episodes of controlled ischemia–reperfusion in other remote organs, i.e. remote ischemic preconditioning^4^. For example, limb^5^ or liver ischemic stimuli^6^, applied prior to coronary artery occlusion, was demonstrated to be associated with reduced infarct size. A number of randomized clinical trials were also conducted and showed the beneficial effect of remote ischemia preconditioning^7,8^. However, despite the overwhelming data indicating the effectiveness of preconditioning-induced cardioprotection, there is concern that the infarct-sparing effect of ischemic conditioning may be abolished or compromised in the diabetic heart^9^. Interestingly, we found that brief ischemic preconditioning of liver reduced the occurrences of myocardial I/R-provoked ventricular arrhythmia in diabetic heart^10^. However, whether this remote liver ischemic preconditioning (RLIPC) could protect diabetic hearts against infarction is incompletely understood.

The exact mechanism underlying the cardioprotective effect of remote ischemic conditioning is unclear. Activation of reperfusion injury salvage kinase (RISK) pathway, or the survivor activating factor enhancement (SAFE) pathways can be involved in ischemic preconditioning and postconditioning^11,12^. We previously reported that RLIPC activated RISK pathway post I/R, specifically, increased ERK1/2^10^, AKT^13^, and GSK-3β^6^ protein phosphorylation. Meanwhile, the inhibition of STAT3, the vital signal molecule in SAFE pathway, abolished the protective action of liver preconditioning^12^. However, whether RLIPC may alter RISK and SAFE pathway in diabetic hearts is unknown.

Therefore, using a left anterior descending coronary artery (LAD) occlusion-induced myocardial I/R rat model, we evaluated the therapeutic efficacy of liver ischemic preconditioning in acute streptozotocin-induced diabetic hearts and reported an underlying molecular mechanism for its protective effect.

## Materials and methods

### Animals

This study was carried out in compliance with the ARRIVE (Animal Research: Reporting of In Vivo Animal Experiments) guidelines. The protocols of animal experiments were approved by the Institutional Animal Care and Use Committee of Sichuan University (2015035A). All experiments were performed according to the recommendations in the Guide for the Care and Use of Laboratory Animals of the National Institutes of Health (NIH Publication 8th edition, 2011). Male rats (Sprague Dawley, 200–250 g body weight, 8 weeks old) were purchased from Dashuo Experimental Animal Research Center (Chengdu, China). The rats were housed in specific-pathogen free environment with a circadian rhythm of 12 h light/12 h darkness and free access to food and water.

### Rat model of type 1 diabetes

Rats received one-time intraperitoneal injection of streptozotocin (STZ, 50 mg/kg) (STZ, Sigma Chemical Co., St. Louis, MO, USA) to develop type 1 diabetes^14^. STZ was dissolved in 0.1 M citrate buffer (pH 4.5). One Touch Ultra Glucose meter was used to measured blood glucose 7 days following STZ injection (Roche, USA). Diabetic rats were defined as rats with glucose levels equal to or more than 20 mmol/L. Rats that did not meet the criteria were excluded.

### Experimental protocol

**Figure 1:**
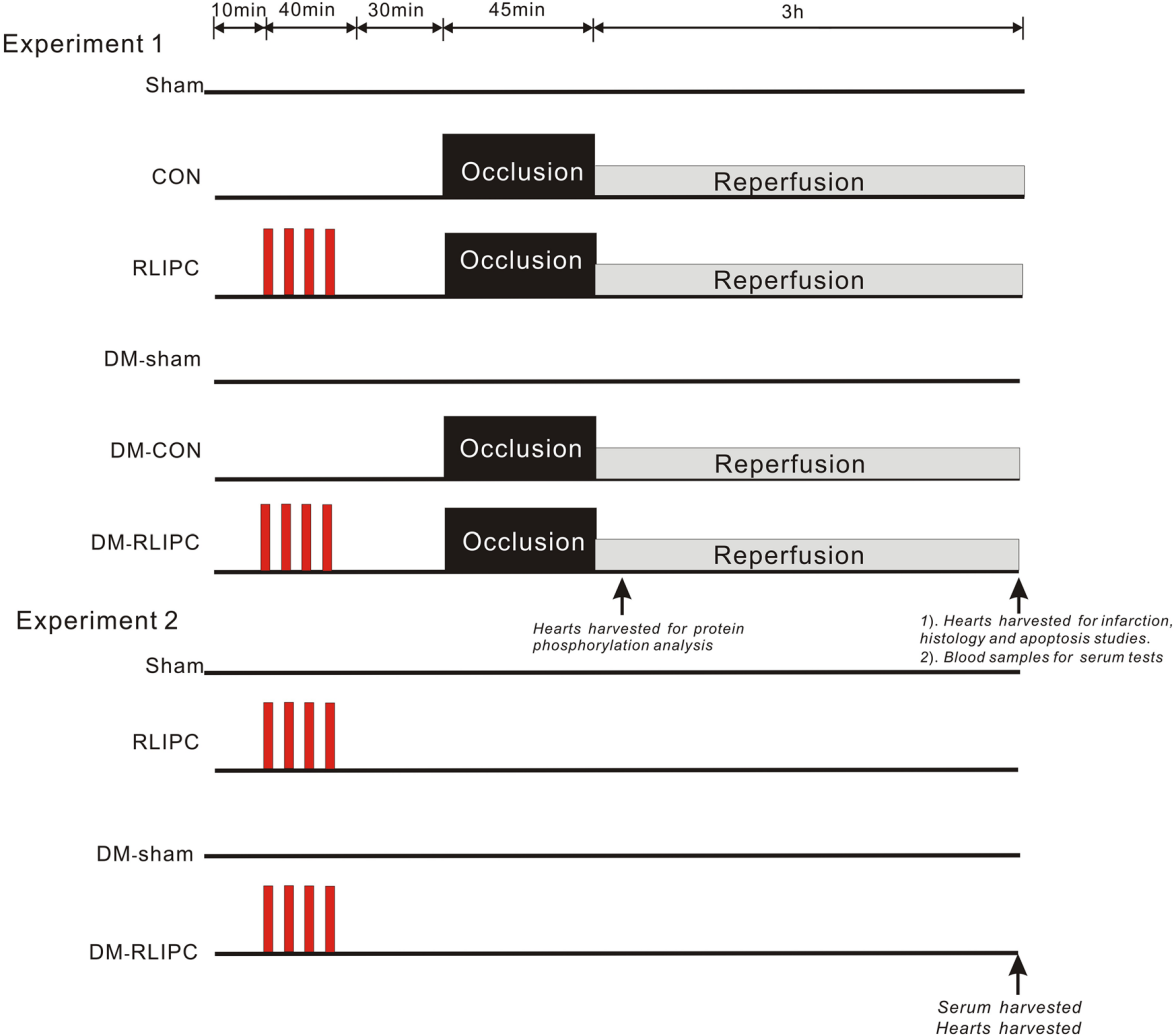
Flowchart of experimental protocol. *Red block:* four cycles of liver ischemic stimuli. *Black block:* duration of left anterior descending (LAD) coronary occlusion. *Grey block*: duration of reperfusion. *Sham:* sham-operated surgery, *CON:* LAD occlusion only, *RLIPC:* remote liver ischemic preconditioning*, DM:* STZ-induced diabetes*.*

The experimental protocols were delineated in Fig. 1. Rats were randomly assigned as follows: (1) sham group (sham) without diabetes: hepatic portal and left coronary artery were isolated with suture placed underneath but not tightened; (2) control group without diabetes (CON): rats were subjected to left anterior descending coronary artery (LAD) occlusion. No hepatic intervention was implemented; (3) CON with RLIPC: rats were subjected to LAD occlusion with pretreatment of hepatic ischemia; (4) sham group with diabetes (DM-sham), hepatic portal artery and LAD were isolated with suture placed underneath but not tightened in diabetic rats; (5) control group with diabetes (DM-CON): diabetic rats had LAD occlusion. No hepatic intervention was implemented; (6) DM-CON with remote liver ischemic preconditioning treatment group (DM-RLIPC): diabetic rats had LAD occlusion with pretreatment of hepatic ischemia. Hearts were taken at 30 min following cardiac reperfusion for protein phosphorylation analysis (Westernblotting analysis) and at 3 h for infarction (TTC staining), histology (hematoxylin and eosin, H&E staining) and apoptosis studies (TUNEL staining). In a parallel study (experiment 2), to test if liver ischemic conditioning caused liver damage, blood samples were obtained at the end of the experiment in sham and RLIPC rat with or without diabetes for the test of serum levels of aspartate aminotransferase (AST) and alanine aminotransferase (ALT).

### Surgical procedure

Rats were anesthetized with sodium pentobarbital (50 mg/kg, intraperitoneal). Anesthesia was monitored by the loss of the corneal reflex. Ligation of LAD was performed as previously reported^6,15^. In brief, after the rat was anesthetized, it was ventilated with a rodent ventilator throughout the experiment (Taimeng, Chengdu, China). After thoracotomy, LAD was exposed and the 6–0 silk was placed under LAD (Ethicon, Somerville, NJ, USA). LAD was occluded for 45 min followed by 180 min of reperfusion. Exhibition of epicardial cyanosis and dyskinesia in the heart was observed as evidence of successful occlusion of LAD. Upon reperfusion, epicardial hyperemic response was seen in LAD region of the heart. Hemodynamic parameters were recorded during the entire experiment (Taimeng, Chengdu, China). Remote liver ischemic preconditioning was done by four cycles of clamping hepatic artery, portal vein and venous trunk for 5 min followed by 5 min reperfusion. At the end of the reperfusion, the rats were euthanized with an overdose of sodium pentobarbital (200 mg/kg, i.p.). LAD was then re-occluded and left ventricle (LV) was filled with 1% Evans blue (Sigma Chemical Co., St. Louis, MO, USA) to show the ischemic area at risk (AAR). Tissue samples in the AAR were then taken and stored in − 80 °C freezer for later protein phosphorylation analysis.

### Hemodynamic analyses

After stabilization, a 20-G catheter (Spacelabs Medical, Inc., Redmond, WA, USA) was inserted into the LV via the right carotid artery. The catheter was then connected to a pressure transducer (Biolap 420F, Taimeng, Chengdu, China) for hemodynamic measurements, such as left ventricular end diastolic pressure (LVEDP), left ventricular systolic pressure (LVSP), and maximum rate of increase/decrease in left ventricular pressure (± dP/dtmax).

### Serum tests

Blood samples were taken at the end of the experiment and the serum was obtained by centrifugation (1000*g*, 10 min, 4 °C). The serum was then frozen at − 20 °C until further analysis. Levels of AST, ALT and α-hydroxybutyrate dehydrogenase (α-HBDH) were measured by an automatic BS-120 biochemical analyzer (Mindray, Shenzhen, China). The serum cardiac troponin I (cTnI) level was detected using a rat Elisa kit according to the manufacturer's instruction (Quanzhou Ruixin Biological Technology Co., Ltd. Fujian, China).

### Determination of myocardial infarct size

Hearts (n = 6–7 per group) were briefly frozen and then cut into transverse slices (2 mm thick) and myocardial infarct size was determined by triphenyltertrazolium chloride (TTC) staining. The heart slices were incubated with 1% TTC (Sigma Chemical Co., St. Louis, MO, USA) in 0.1 M phosphate buffer (pH 7.4) for 20 min at 37 °C. Tissues were then fixed in 10% formalin at room temperature overnight. Infarcted myocardial tissues within AAR were unstained (white) and non-infarcted areas were stained red. They were carefully separated and weighed. Infarct size was presented as a percentage of the AAR.

### Heart tissue collection

At the end of the experiment, AAR regions were identified and were immersed in 10% formaldehyde solution, followed by dehydration in a separated group of hearts. These hearts were embedded in paraffin and sliced into 5 μm thick consecutive sections parallel to the atrioventricular groove. Heart sections were mounted on glass slides prior to H&E staining or apoptosis measurements.

### Pathological evaluation

Myocardial pathological scores were determined based on a modified numerical scoring system^15^ according to: (1) the severity of myocardial damage (i.e. myofibril degeneration, oedema, or subendocardial haemorrhage) with 0 indicating normal, 1 mild, 2 moderate and 3 significant; (2) the distribution of myocardial damage with 0 indicating normal, 1 focal, 2 multifocal and 3 diffuse. A mean score was calculated for each heart in a double-blinded manner. The myocardial pathological scoring performed by two researchers who were blinded to the study, n = 5 per group.

### TUNEL staining

The heart sections (n = 5–6 per group) were stained by the In Situ Cell Death Detection Kit (Roche Diagnostics, Indianapolis, IN, USA) according to the manufacturer’s instructions. TUNEL-positive nuclei were stained green. 15 separate fields were chosen randomly per heart section blindly. The apoptotic index in the left anterior ventricular wall was expressed as a percentage of the total nuclei population. A fluorescent microscope (Nikon Instruments Inc.) was used for observation. Images were analyzed with Image-pro plus (Media Cybernetics, Inc., Carlsbad, CA, USA).

### Western blotting

Heart tissue isolated from the AAR was used for Westernblotting analysis, n = 5–6 per group. Samples were homogenized in lysis buffer consisting of 150 mM NaCl, 50 mM Tris–HCl (pH 7.4), 0.25% sodium deoxycholate, 1% NP-40, 1 mM EDTA, and phosphatase and protease inhibitor cocktails (Sigma Chemical Co., St. Louis, MO, USA). The homogenates were then centrifuged at 4 °C for 10 min at 10,000×*g*. Protein concentration was determined using BCA assay kit (Pierce, Rockford, IL, USA). Sample were separated on 12% SDS-PAGE gel (15 μg/well). The protein bands were then transferred onto nitrocellulose membranes (VWR, Batavia, IL, USA). The membranes were then blocked for 1 h and incubated at 4 °C overnight with the following primary antibodies: phosphorylated extracellular signal-regulated kinase 1/2 (ERK1/2) (Thr202/Tyr204), total ERK1/2, phosphorylated glycogen synthase kinase-3β (Ser9) (p-GSK-3β Ser9), total-GSK-3β Ser9, phospho-Akt (Ser473, p-Akt), total Akt, phosphorylated STAT3 (Tyr705) (p-STAT3), and total STAT3, phosphorylated STAT5 (Tyr694) (p-STAT5), and total STAT5 (all: rabbit, 1:1000, from Cell Signaling Technology, Danvers, MA, USA). We used horseradish peroxidase (HRP)-conjugated goat anti-rabbit IgG as secondary antibody. The target bands were detected using a chemoiluminescence ECL (Millipore, Billerica, MA, USA) and visualized using AmershamImager 600 (GE healthcare, Little Chalfont, UK). The images were then analyzed with ImageJ Data Acquisition Software (National Institutes of Health, Bethesda, MD, USA).

### Statistical analysis

All data were presented as mean ± standard error of the mean (SEM). Statistical analyses were performed using SPSS 13.0 software for Windows (SPSS Inc., Chicago, IL, USA) or Graphpad Prism 5 software (GraphPad Software, Inc. La Jolla, CA, USA). Two-way repeated-measures ANOVA was used to analyze hemodynamics data. One-way ANOVA followed by Newman–Keuls test was used for multiple group comparison. Statistical significance was set as *p* < 0.05 (two-tailed).

## Results

### Confirmed phenotype of DM rats

**Figure 2:**
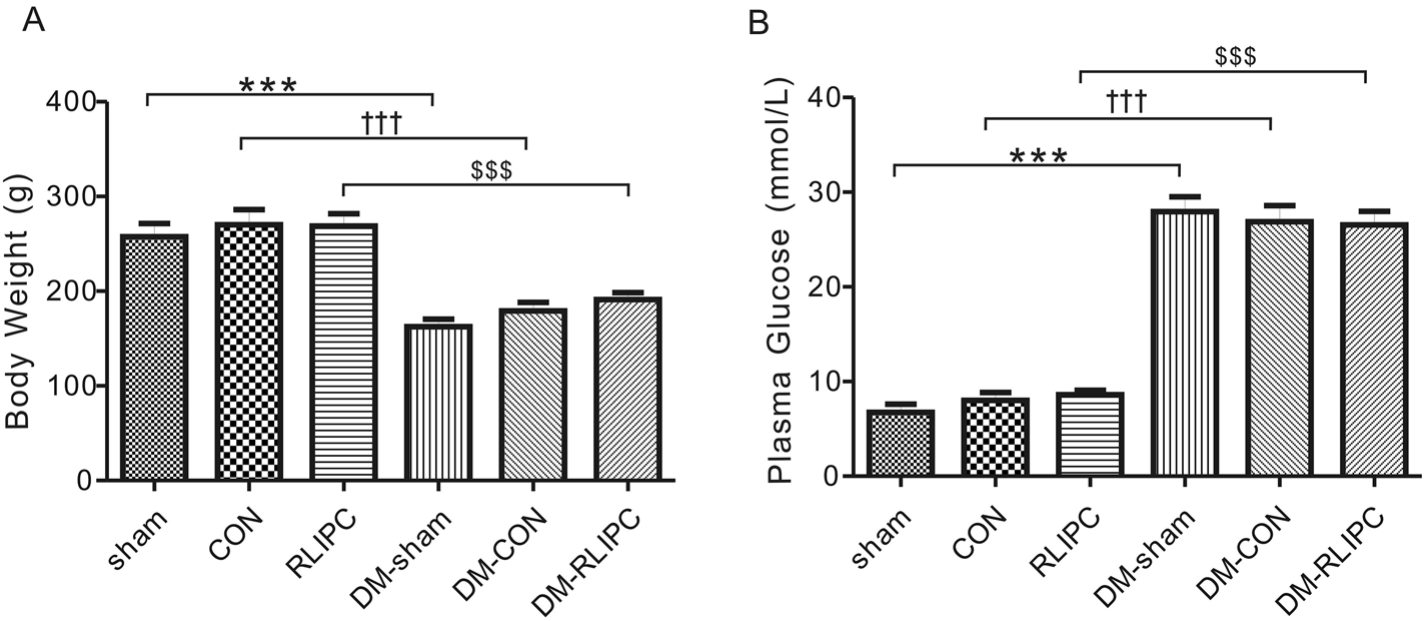
Body weight and plasma glucose after STZ treatment. (**A**) Body weight (n = 7–10); (**B**) plasma glucose (n = 6–10). Sham, sham surgery; CON, LAD ligation; RLIPC, remote liver ischemic pre-conditioning; DM: STZ-induced diabetes. Data presented as mean ± SEM. ***p < 0.001 compared with sham rats, ^†††^*p* < 0.001 compared with CON, ^$$$^*p* < 0.001 compared with RLIPC rats (by one-way ANOVA).

Our experimental protocol successfully induced diabetes (DM) in the rats by single intraperitoneal administering of STZ. After 7 days, DM rats showed a 30% decrease in body weight (all *p* < 0.001, Fig. 2A) and hyperglycemia with doubled or tripled blood glucose level (all *p* < 0.001, Fig. 2B) when compared to rats without STZ injection in sham, CON and RLIPC group.

### Remote liver ischemic preconditioning reduces myocardial infarct size

**Figure 3:**
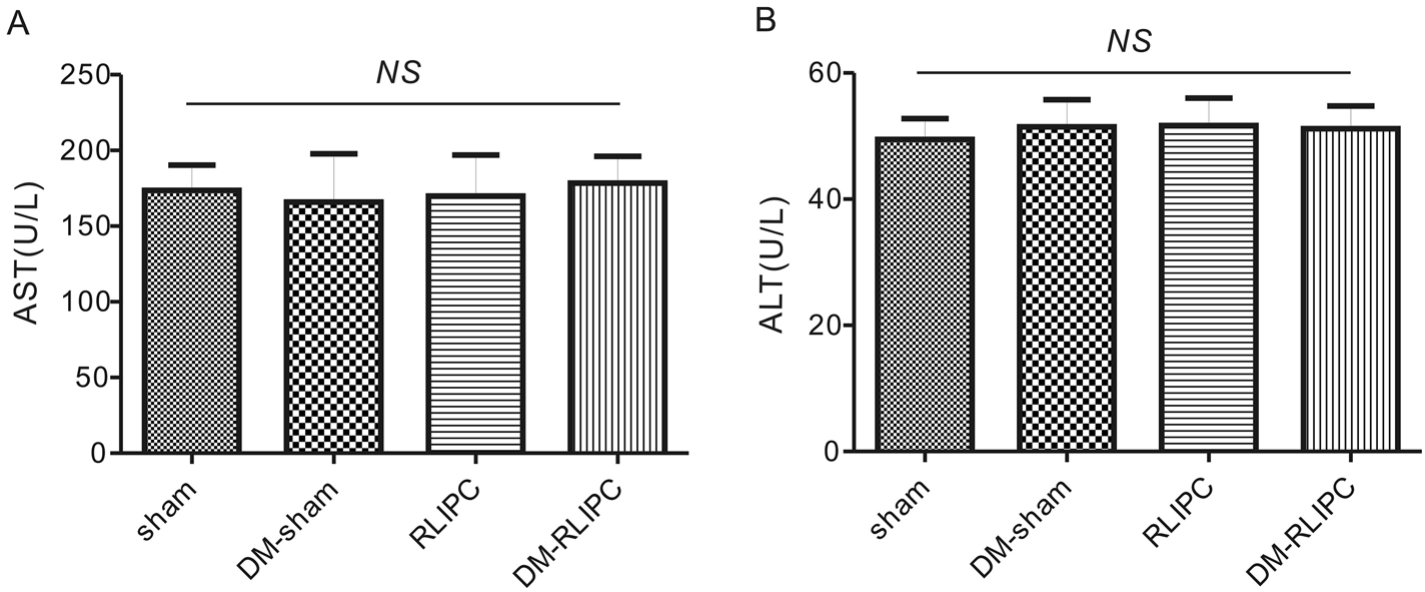
Remote ischemic preconditioning did not cause liver injury. (**A**) Plasma AST (n = 5–6); (**B**) plasma ALT (n = 5–6). Sham, sham surgery; RLIPC, remote liver ischemic preconditioning; DM: diabetic rats. Data presented as mean ± SEM. NS: non-significant between groups (*p* > 0.05, by one-way ANOVA).

**Figure 4:**
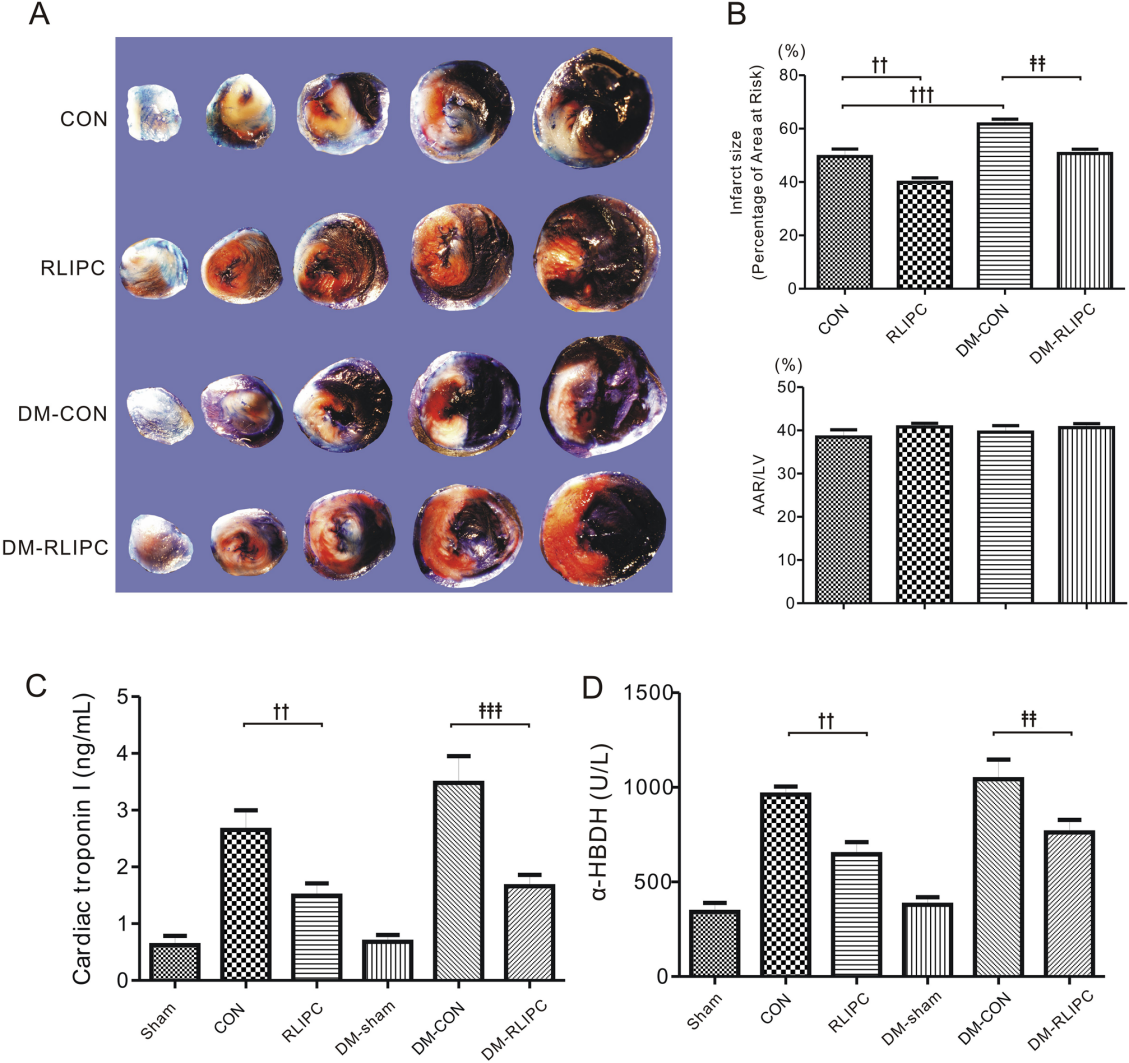
Remote ischemic preconditioning alleviated myocardial infarction. (**A**) Representative sections of triphenyltetrazolium chloride (TTC)-stained heart subjected to 45 min myocardial ischemia followed by 3 h of reperfusion. Sham, sham surgery; CON, LAD ligation; RLIPC, remote liver ischemic preconditioning; DM: diabetic rats. (**B**) Quantification of myocardial infarct size expressed as a percentage of left ventricular (LV) area at risk (AAR) (top) and AAR expressed as a percentage of LV area (bottom). Data were presented as mean ± SEM; n = 6–7 each group. ^††^*p* < 0.01 and ^†††^*p* < 0.001 compared with CON; and ^‡‡^*p* < 0.01 compared with DM-CON (by one-way ANOVA). (**C**) Post-reperfusion injury mean serum levels of cardiac troponin I (cTnI) in rats subjected to 45 min of left anterior descending artery occlusion followed by 3 h of reperfusion. n = 6–8 each group. ^††^*p* < 0.01 compared with CON; and ^‡‡‡^*p* < 0.001 compared with DM-CON (by one-way ANOVA). (**D**) Post-reperfusion injury mean serum levels of *α*-hydroxybutyrate dehydrogenase (*α*-HBDH). n = 6–8 each group. ^††^*p* < 0.01 compared with CON; and ^‡‡^*p* < 0.01 compared with DM-CON (by one-way ANOVA).

We first investigated if RLIPC caused liver injury. We found that there was no significant difference in serum levels of AST (Fig. 3A) and ALT (Fig. 3B) among sham and RLIPC-treated rats with or without diabetes (all *p* > 0.05). This result suggested that RLIPC did not cause liver injury. After RLIPC, myocardial I/R was induced. Infarct size of the diabetic rats increased approximately 24% when compared with non-diabetic rats after myocardial I/R as a result of LAD occlusion and re-opening (61.74% ± 1.82% vs. 49.58% ± 2.78%, *p* < 0.001) (Fig. 4A,B). This suggests that diabetic state aggravates the I/R injury caused by LAD ligation. Interestingly, we found that RLIPC resulted in a 20% reduction of cardiac infarct size when compared to non-RLIPC group in both non-diabetic rat (39.91% ± 1.66% in RLIPC vs. 49.58% ± 2.78% in CON, *p* < 0.01) and DM rats (50.70% ± 1.59% in DM-RLIPC vs. 61.74% ± 1.82% in DM-CON, *p* < 0.01) (Fig. 4A,B), suggesting RLIPC effectively limited the infarct size in diabetic and non-diabetic rats. Meanwhile, we did not see any differences in the ratio of the AAR to the LV among groups, indicating similar areas affected by LAD ligation (Fig. 4B, *p* > 0.05). The levels of cardiac troponin I (cTnI) and α-HBDH can reflect the degree of myocardial damage. We found in our study that myocardial reperfusion caused an elevation of serum cTnI and α-HBDH level, suggesting that reperfusion-related myocardial injury occurred during reperfusion period. However, RLIPC treated diabetic or non-diabetic rats exhibited lower serum levels of cTnI or α-HBDH as compared to corresponding diabetic or non-diabetic controls (Fig. 4C,D, *p* < 0.01 or *p* < 0.001).

**Figure 5:**
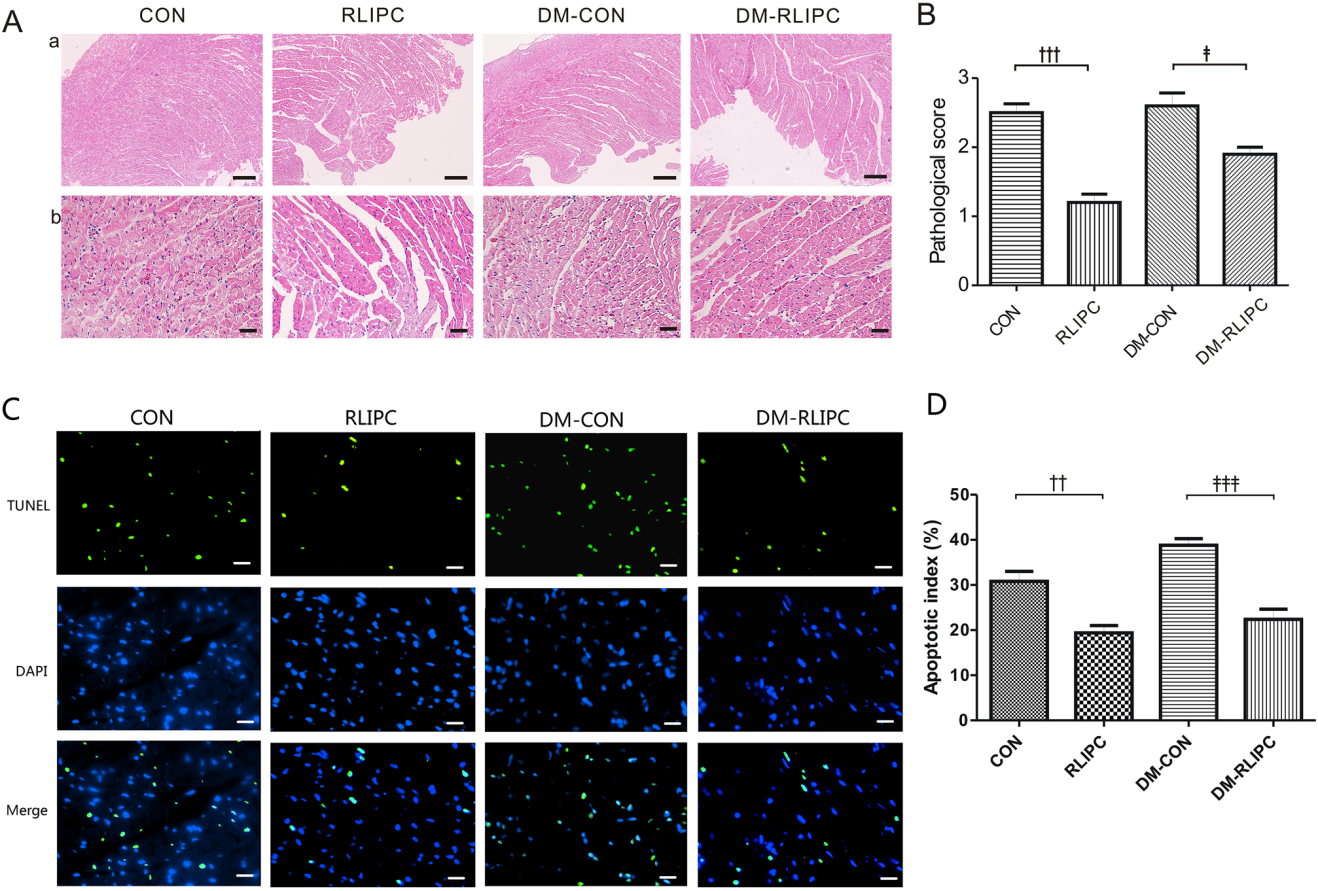
Effect of RLIPC on morphological changes post-myocardial ischemia/reperfusion. (**A**) Representative (of n = 5 rats/group) H&E stained heart sections are shown. Panel (a) scale bars: 100 μm; panel (b) scale bars: 10 μm. Sham, sham surgery; CON, LAD ligation; RLIPC, remote liver ischemic preconditioning; DM: diabetic rats. (**B**) Morphological evaluation of myocardium damage in each heart (n = 5 mice per group). The severity of myocardial damage was graded from 0 to 3 (see the ‘Methods’ section). Data were presented as mean ± SEM; ^†††^*p* < 0.001 compared with CON; and ^‡^*p* < 0.05 compared with DM-CON (by one-way ANOVA). (**C**) Representative TUNEL-stained heart sections. TUNEL-positive (green) cardiomyocytes were identified as apoptotic cells. Scale bars: 10 μm. (**D**) Bar graph showing the average percentage of TUNEL-positive nuclei in the ischemic regions (n = 5–6 rats per group). ^††^*p* < 0.01 compared with CON; and ^‡‡‡^*p* < 0.001 compared with DM-CON (by one-way ANOVA).

We also evaluated the cardiac injury using the pathological scoring system. Consistent with TTC staining results, RLIPC treated non-diabetic or diabetic rats had reduced pathological score when compared to non-diabetic or diabetic control rats, respectively (*p* < 0.001 or *p* < 0.05, Fig. 5A,B). After 3 h of reperfusion, TUNEL-positive nuclei were found within the left ventricles, however, non-diabetic control rats (30.8 ± 2.2%) had markedly more apoptotic nuclei than did RLIPC-treated non-diabetic rats (19.4 ± 1.6%, *p* < 0.01). In the meantime, diabetic control hearts (38.8 ± 1.4%) exhibited more severe apoptosis when compared to RLIPC-treated diabetic hearts (22.4 ± 2.2%, *p* < 0.001, Fig. 5C,D).

### Hemodynamic measurements

The time course of hemodynamics was shown in Table 1. Systemic hemodynamics were comparable among each group under baseline conditions (*p* > 0.05). LVSP (*p* = 0.002), dP/d*t*_max_ (*p* = 0.001), LVEDP (*p* = 0.003) and − dP/d*t*_max_ (*p* = 0.001) were significantly different among CON, RLIPC, DM-CON and DM-RLIPC group during the 3 h of reperfusion. There were significant interactions between groups and the time course for LVSP (*p* = 0.011), dP/d*t*_max_ (*p* = 0.028), LVEDP (*p* = 0.003) and − dP/d*t*_max_ (*p* = 0.001). Recovery of cardiac function was significantly better in RLIPC group in both non-diabetic and DM rats when compared to CON group in terms of the above-mentioned parameters (*p* < 0.01 for all).

**Table 1:** The effect of RLIPC on hemodynamics.

Variable	Baseline	Reperfusion		
		1 h	2 h	3 h
**LVSP (mmHg)**				
CON	135.3 ± 10.1	104.1 ± 11.2***	98.8 ± 5.2***	85.9 ± 11.6***
RIPC	137.5 ± 4.0	110.8 ± 6.3**	111.5 ± 10.6***^#^	104.3 ± 6.7***^##^
DM-CON	139.8 ± 13.9	101.7 ± 7.2***	96.0 ± 4.7***	91.0 ± 9.5***
DM-RLIPC	136.7 ± 8.0	110.0 ± 4.8***^†^	112.2 ± 6.8***^††^	105.5 ± 5.8***^††^
**LVEDP (mmHg)**				
CON	− 5.3 ± 1.9	1.0 ± 0.3***	3.0 ± 0.8***	5.7 ± 1.6***
RLIPC	− 5.1 ± 2.0	− 1.2 ± 0.9**^#^	0.7 ± 0.2***^##^	2.2 ± 0.9***^###^
DM-CON	− 6.3 ± 1.8	1.4 ± 0.8***	3.3 ± 2.1***	5.4 ± 0.8***
DM-RLIPC	− 5.6 ± 1.9	− 2.7 ± 1.6*^†^	− 0.2 ± 0.2**^†^	2.9 ± 1.1***^††^
**dp/dtmax (mmHg/ms)**				
CON	5.1 ± 0.6	2.9 ± 0.6***	2.3 ± 0.9***	1.6 ± 0.6***
RLIPC	5.3 ± 1.1	3.7 ± 1.0*	3.5 ± 0.7**^#^	3.4 ± 0.6***^###^
DM-CON	5.6 ± 0.7	2.7 ± 0.7***	2.4 ± 1.0***	1.5 ± 0.6***
DM-RLIPC	5.4 ± 1.0	3.8 ± 1.1*	3.9 ± 0.8*^†^	3.2 ± 0.3***^†††^
**− dp/dtmax (mmHg/ms)**				
CON	− 4.9 ± 0.5	− 2.8 ± 0.5***	− 2.2 ± 0.3***	− 1.4 ± 0.7***
RLIPC	− 5.2 ± 1.0	− 4.1 ± 1.2***^#^	− 3.3 ± 0.9**^#^	− 3.1 ± 0.7**^##^
DM-CON	− 4.8 ± 0.8	− 2.2 ± 0.9***	− 2.1 ± 0.9***	− 1.3 ± 0.6***
DM-RLIPC	− 5.3 ± 0.9	− 4.0 ± 0.4*^††^	− 3.5 ± 0.8**^†^	− 3.4 ± 0.5**^†††^

Data are expressed as mean ± SEM. *CON:* LAD occlusion only, *RLIPC:* remote liver ischemic preconditioning*, DM: *STZ-induced diabetes. LVSP = left ventricular systolic pressure, LVEDP = left ventricular end-diastolic pressure; ± dP/dtmax = maximum rate of increase/decrease in left ventricular pressure. n = 6 in each group.

*p < 0.05, **p < 0.01, ***p < 0.001 versus baseline, ^#^*p* < 0.05, ^##^*p* < 0.01, ^###^*p* < 0.001 versus CON, ^†^*p* < 0.05, ^††^*p* < 0.01, ^†††^*p* < 0.001 versus DM-CON.

### RISK and SAFE pathway protein phosphorylation

**Figure 6:**
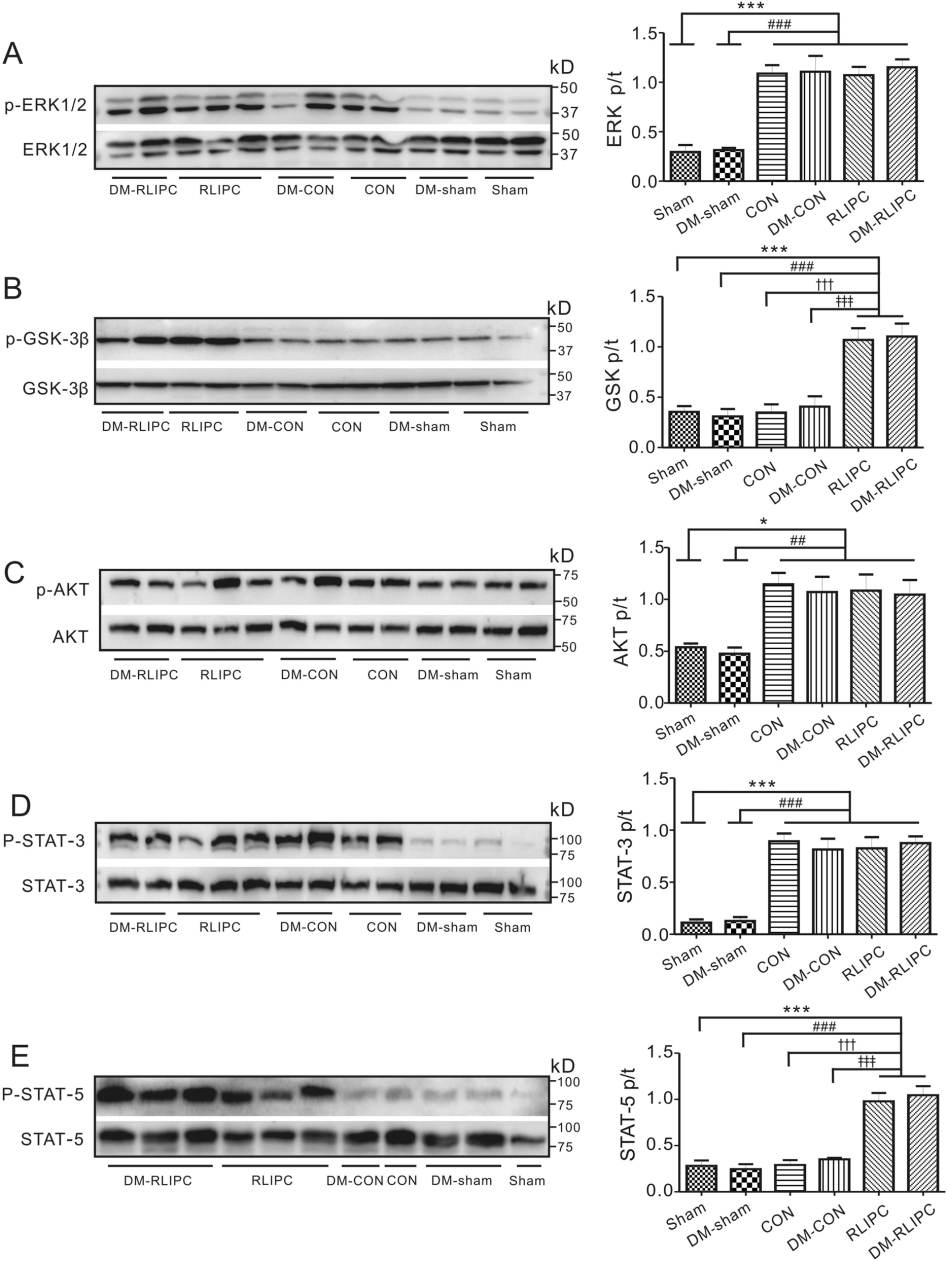
RLIPC stimulated GSK-3β and p-STAT-5 phosphorylation in both non-diabetic and diabetic rats. Representative Western blots (left) and quantification (right) of p-ERK1/2 (**A**), p-GSK-3β (**B**), p-AKT (**C**), p-STAT-3 (**D**) and p-STAT-5 (**E**) protein band densities (normalized to total protein, respectively) in sham, CON and RLIPC group in both non-diabetic and diabetic rats. Sham, sham surgery; CON, LAD ligation; RLIPC, remote liver ischemic preconditioning; D: diabetic rats. (n = 5–6, each group), data were presented as mean ± SEM. *p < 0.05 and ***p < 0.001 compared with Sham. ^#^*p* < 0.05 and ^###^*p* < 0.001 compared with DM-sham. ^†††^*p* < 0.001 compared with CON. ^‡‡‡^*p* < 0.001 compared with DM-CON (by one-way ANOVA).

ERK phosphorylation is reported to be associated with cardiac pathophysiological process like myocardial infarction. We found that LAD ligation significantly increased ERK1/2 phosphorylation by more than 2.5 folds in both non-diabetic (*p* < 0.001) and DM rats (*p* < 0.001) (Fig. 6A). However, RLIPC did not alter the pattern of increased ERK phosphorylation after LAD ligation in both non-diabetic and DM rats (Fig. 6A). This suggested that ERK phosphorylation was not associated with RLIPC-induced cardioprotection in diabetic hearts. We previously found GSK-3β phosphorylation was associated with cardiac protection offered by liver ischemic conditioning^6^. As expected, we again showed that RLIPC significantly increased GSK-3β phosphorylation by 2 folds when compared to CON rats (*p* < 0.001), and the same effect was also observed in DM rats (*p* < 0.001) (Fig. 6B). This suggested increased GSK phosphorylation caused by RLIPC was not affected by diabetic state. AKT signaling pathway was reported to be associated with cardiac injury caused by myocardial infarction. Consistently, we also found that AKT phosphorylation increased by 110% in the non-diabetic CON when compared to non-diabetic sham ones (*p* < 0.05). However, RLIPC could not further increase the phosphorylation levels of AKT in both non-diabetic and DM rats when compared with their corresponding non-RLIPC controls (Fig. 6C).

The signal transducers and activators of transcription (STAT) functions as regulators of cellular stress. We next investigated if STAT signaling was involved in the cardiac protection from RLIPC. We found that STAT3 phosphorylation was significantly increased by 5–7 folds in the DM-CON and non-diabetic CON groups compared to diabetic sham (*p* < 0.001) and non-diabetic sham (*p* < 0.001) groups, respectively. RLIPC did not alter the expression pattern of STAT3 phosphorylation after LAD ligation (Fig. 6D), suggesting STAT3 signaling pathway is not associated with RLIPC-induced cardioprotection. Surprisingly, although STAT5 phosphorylation was similar between rats with LAD ligation and sham surgery, we found that RLIPC increased STAT5 phosphorylation by more than two folds compared to non-RLIPC-treated control rats in both non-diabetic (2.4 fold, *p* < 0.001) and DM groups (twofold, *p* < 0.001), indicating that STAT5 signaling plays a role in the cardioprotective effect of RLIPC in both non-diabetic and diabetic rat hearts (Fig. 6E).

## Discussion

The present data suggests that pretreatment with liver ischemic preconditioning prior to a 45 min LAD occlusion and a subsequent 3 h reperfusion reduced myocardial injury in STZ-induced diabetic rat hearts, as shown by reduced infarct size, decreased apoptosis and pathological score. To our knowledge, this is the first study reporting the results of using RLIPC in a diabetic myocardial I/R model. Our data demonstrate that increased glycogen synthase kinase-3β (GSK-3β) and STAT-5 phosphorylation may be associated with RLIPC treatment in diabetic hearts.

### Diabetes and myocardial protection

DM is a common metabolic disorder, characterized by hyperglycemia, hyperlipidemia, and hypoinsulimia. Patients with DM have increased risk of coronary artery disease and myocardial infarction^24^. Furthermore, diabetic patients exhibit a higher sensitivity to myocardial reperfusion-induced injury^16^, as such, it is more difficult for diabetic patients to recover from heart attack after pharmacological or mechanical reperfusion strategies including fibrinolytic therapy or percutaneous transluminal coronary intervention (PCI)^17^. Our study revealed that myocardial damage caused by reperfusion injury was more severe in diabetic rats than that in non-diabetic rats. This suggests that hyperglycemia may have greater adverse impact on cardiomyocytes in response to ischemia and reperfusion stimuli. Therefore, effective therapeutic approaches and novel targets are required to rescue reperfusion-injured myocardium in diabetic state.

Since remote ischemic conditioning was found beneficial to the recovery after myocardial infarction, multiple clinical trials have conducted to evaluate the effect of remote limb ischemic conditioning in patients with myocardial infarction. Botker and colleagues showed that remote limb ischemic conditioning applied during myocardial infarction period before hospital admission increased salvaged area of myocardium^7^. Other clinical studies have also confirmed the beneficial effect of limb ischemic conditioning during cardiac surgery, elective PCI, and acute myocardial infarction^4^. Additionally, liver is the biggest metabolic organ in the body that remote ischemic conditioning can be applied. Compared with limb ischemic conditioning, which has been studied intensively, the effect of liver ischemic conditioning was largely unknown. We and others have reported that RLIPC reduced infarct area in non-diabetic hearts in vivo^10^ or ex vivo^18^. More recently, our laboratory demonstrated the existence of anti-arrhythmic effect of RLIPC post myocardial I/R in diabetic heart^14^. In the current study, we tested the efficacy of RLIPC in STZ-injected rats whose beta pancreatic cells were destroyed leading to type I diabetes phenotype. To the best of our knowledge, RLIPC-induced infarct-sparing effects has never been tested in type 1 diabetes models. The current results demonstrated that pretreatment of liver ischemic stimuli before sustained myocardial ischemia limits infarction post-I/Rin diabetic rats. However, our results contrast with reports concerning that efficacy of ischemic conditioning could be attenuated, or may even completely lost in diabetes^19–21^. This discrepancy may be explained by the possible interactions between anti-diabetic medication and remote ischemic conditioning, different protocol designs of conducting ischemia cycles and observing different primary outcomes, as well as differences in animal species.

### RISK/SAFE pathway in diabetes

Although efforts have been made trying to find new signaling targets contributing to the remote ischemic preconditioning-induced anti-infarction against myocardial I/R injury, little is known about the potential role of RLIPC in cardioprotection in diabetic hearts. Reperfusion injury salvage kinase (RISK) pathway, first described by Yellon et al.^11^ has been demonstrated to be involved in remote ischemic conditioning in multiple studies^22^. RISK pathway includes two major kinases cascades: the p42/p44 extracellular signal-regulated kinases (ERK1/2) and kinase B (AKT), all of those are pro-survival protein kinases, responsible for cell proliferation, transcription and survival^11^. The RISK pathway can be activated in response to stress such as ischemia–reperfusion, initiating phosphorylation of a wide array of intracellular targets, resulting in modification of protein synthesis. It has been shown that either ischemic conditioning or pharmaceuticals could induce ERK1/2 or AKT phosphorylation, thus ultimately reduced myocardial infarct size^23,24^. We previously found that RLIPC protected hearts against sudden cardiac death via activation of ERK1/2 pathway^10^. Meanwhile, we also observed increased phospho-AKT levels in the brain of RLIPC rats compared to controls^13^. However, results from literatures regarding whether AKT can be activated (phosphorylated) in diabetic myocardium are conflicting. Although some studies have shown that the failure of cardioprotection by ischemic conditioning in diabetes has been attributed to the decreased activation of AKT signaling molecules^9,16^, other reports have indicated that AKT phosphorylation level could be increased^25^ in diabetic heart when compared to their non-diabetic controls after I/R injury. The AKT activation profile may be dynamic during reperfusion period, therefore, we did not observe altered AKT phosphorylation between control and DM hearts at 30 min following cardiac reperfusion, which is consistent with our previous findings regarding RLIPC-induced anti-arrhythmic activity^14^. Furthermore, our current study extends these above findings by showing that pretreatment with liver I/R stimulus prior to LAD occlusion did not enhance ERK1/2 or AKT phosphorylation in diabetic or non-diabetic hearts. It seems that the infarction sparing effect of RLIPC against myocardial I/R injury is independent of these two signaling cascades. However, GSK-3β, the vital component of the RISK pathway, is an essential regulator of survival in cardiac myocytes, thus is involved in the pathogenesis of myocardial I/R injury. The phosphorylation within the amino-terminal domain of GSK-3β at Ser9 results in the inhibition of GSK-3 kinase activity^26^ and inactivation therefore is cardioprotective^27^. Prior studies provide evidence that ischemic conditioning can stimulate GSK-3β phosphorylation^28^. We found in our previous study that RLIPC caused GSK-3β phosphorylation in normal hearts post I/R^6^, our current study extends these findings by showing that RLIPC exerted cardioprotection via increasing phosphorylation of ventricular GSK-3β in diabetic hearts compared with non-RLIPC treated diabetic hearts post I/R. Our data are consistent with previous studies showing that pretreatment with GSK-3β inhibitors prior to myocardial ischemia produced cardioprotection in diabetic hearts^29^.

The survivor activating factor enhancement (SAFE) pathways, another cell survival pathway independent of the RISK pathway has been shown to be associated with I/R injury^30,31^. It involves the activation of signal transducer and activator of transcription 3 (STAT3) and 5 (STAT5). Previous studies have shown that STAT3 or STAT5 phosphorylation at reperfusion was increased with remote preconditioning in various animal models^12,32^. Accordingly, the protective effect of preconditioning can be blocked with the administration of STAT inhibitors^33^. We previously showed that increased phospho-STAT3 and STAT5 levels were found in the RLIPC-treated rat lungs and the administration of STAT inhibitor could effectively block the pulmonary protection offered by RLIPC^12^. However, it contrasts with our current findings that STAT5 phosphorylation, not STAT3, played a role in the protective effect of RLIPC in both non-diabetic and diabetic rats. This suggested that activation of SAFE pathway may be organ specific. Taken together, RLIPC induced infarction sparing effect in diabetic and non-diabetic hearts may share a similar mechanism involving activation of GSK-3β and STAT-5 signaling pathways.

The underlying mechanisms through which short episodes of brief I/R stimuli in remote organ or tissue could transfer a protective signal to the target organ are not fully elucidated. It has been proposed that a number of circulating factors may play an important role in this protective phenomenon. Erythropoietin (EPO), an important hormone produced by the kidney, is a major regulator of red blood cell generation. It has been shown that treatment with EPO could limit the infarct size^34^, inhibit the I/R-induced myocardial inflammatory response^35^, and promote survival of cardiomyocytes by enhanced phosphorylation of GSK-3β signaling^36^. EPO is a crucial mediator of remote ischemic preconditioning. The release of EPO could confer cardioprotection during RIPC and inhibition of EPO-related signaling pathways attenuated the RIPC-induced infarct size sparing effect^37^. Meanwhile, the cardioprotective effect of RIPC was absent in rats with renal failure, indicating that insufficient EPO release from kidney may blunt RIPC-induced protection^38^. In addition, microRNA has also been proposed to play a role in the infarct-sparing effect of RIPC. Li et al. found that I/R injury decreased myocardial expression of miR-144, whereas limb I/R conditioning increased serum concentrations of miR-144 and caused elevated expression of miR-144 in the heart. Meanwhile, treatment with miR-144 had both early and delayed cardioprotection, while the inhibition of miR-144 abolished the beneficial effect conferred by RIPC^39^. These findings suggest a vital role of circulating factors in mediating RIPC-induced cardioprotection. Future clinical trials and cohort studies are needed to identify and confirm the efficacy of RIPC.

## Limitations and conclusions

Our study has several limitations. First, animal model of type 1 diabetes (streptozotocin induced) was used in the current study, rather than a high-fat diet induced type of diabetes (type II), the latter may better mimic human metabolite signature, characterized by insulin resistance and hyperinsulinemia. Thus, it is not clear if RLIPC may exert cardioprotection against I/R injury on type 2 diabetes. Second, our protocol had fixed preconditioning cycle of liver ischemia and reperfusion stimuli, therefore, it is not certain if the number of ischemic cycles and duration of ischemic period have dose–response relationship in terms of the cardioprotective effect of RLIPC. Third, we observed the infarct sparing effect of RLIPC using a model of acute myocardial I/R injury, whether RLIPC produces long-term cardioprotection against cardiac fibrosis deserves further determination. Fourth, diabetes may desensitize, remodel or even shift other signaling molecules. We only determine several signaling molecules in RISK and SAFE pathway, it is possible that other molecules and signaling cascades may also be involved in the cardioprotective effect of RLIPC. In addition, our study did not investigate the interaction between pathways, such as the crosstalk between RISK and SAFE pathway. Therefore, future attention will be focused on functional studies so as to identify the relationship between the cardioprotective effect and those altered molecules. Finally, our animal model is limited in that only significant hyperglycemia was present. Nevertheless, given the importance of hyperglycemia in aggravating I/R injury in the heart, our results from this model do provide relevant understanding on RLIPC mediated myocardial protection during ischemia and reperfusion.

In conclusion, liver ischemic preconditioning exerts strong cardioprotective effects in diabetic heart post I/R injury, as evidenced by decreased infarction size, improved cardiac function and alleviated cardiac damage. This RLIPC-induced cardioprotection is mediated by the activation of GSK-3β and STAT-5 signaling pathways.

## Supplementary information

Supplementary Information.
