# Supplementary material for: Remote liver ischemic preconditioning attenuates myocardial ischemia/reperfusion injury in streptozotocin-induced diabetic rats
Source: Sci Rep. 2021 Jan 21;11:1903. doi: 10.1038/s41598-021-81422-1 (PMC7820418; doi:10.1038/s41598-021-81422-1)

**Remote Liver Ischemic Preconditioning Attenuates Myocardial Ischemia/Reperfusion Injury in streptozotocin-induced Diabetic Rats**

Xinhao Liu^1^, Hui Chen^2^,Zhibing Yan^2^, Lei Du^1^, Dou Huang^1^, WeiDong Gao^3^, Zhaoyang Hu^2*^

^1^Department of Anesthesiology, West China Hospital, Sichuan University, Chengdu, Sichuan, China.

^2^Laboratory of Anesthesiology & Critical Care Medicine, Translational Neuroscience Center, West China Hospital, Sichuan University, Chengdu, Sichuan, China

^3^Department of Anesthesiology and Critical Care Medicine, Johns Hopkins University School of Medicine, Baltimore, Maryland, USA. 21287

*To whom correspondence should be addressed: Dr. Zhaoyang Hu, zyhu@hotmail.com

The phosphorylation levels of each **protein were measured, and normalized to** the respective **total protein** loaded in the corresponding lane in the same membrane to reflect the relative ratio of phosphorylated protein densities **to total** protein densities.

marker information:


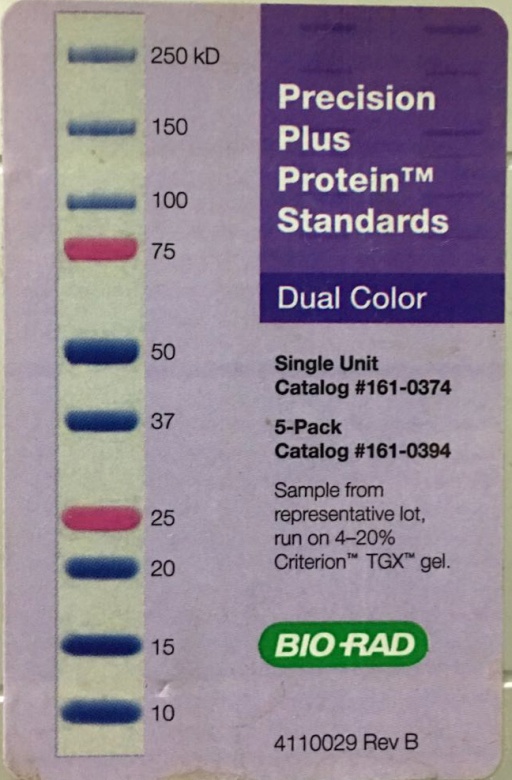


Figure 6A


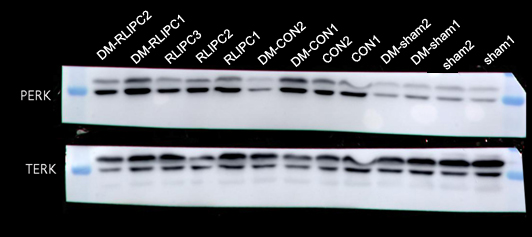


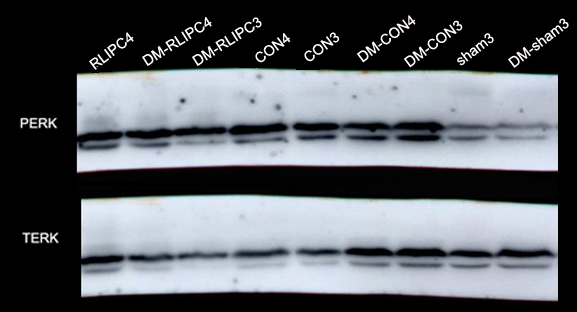


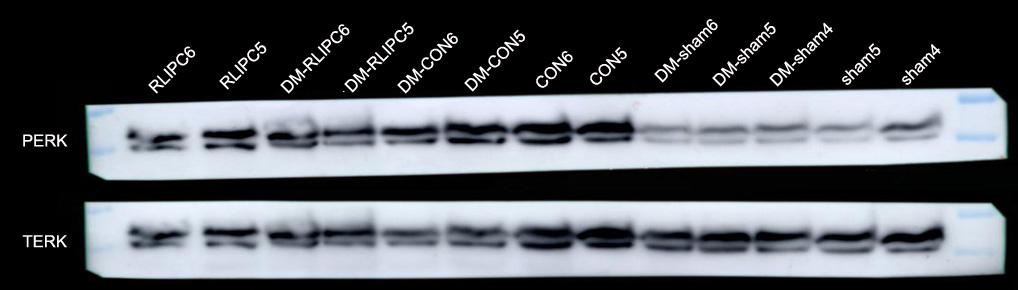


Figure 6B


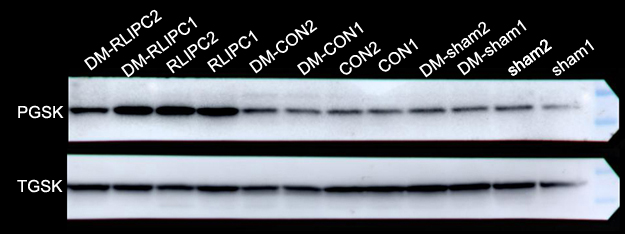


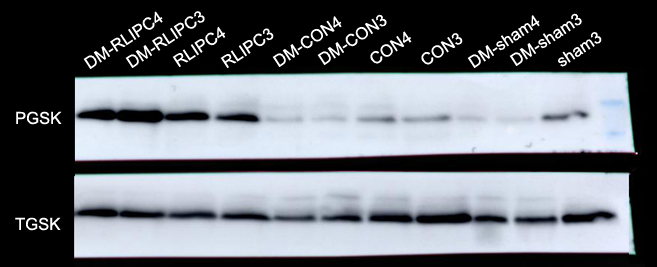


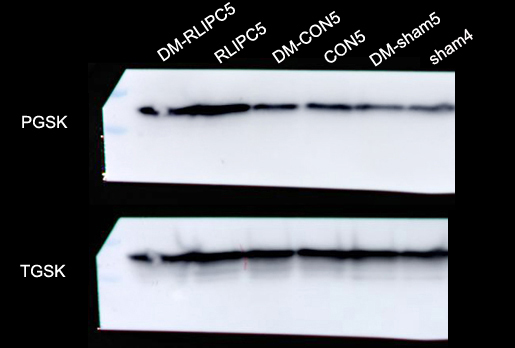


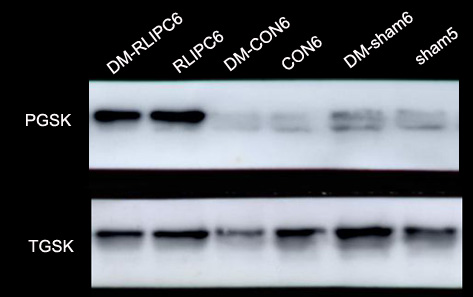


Figure 6C


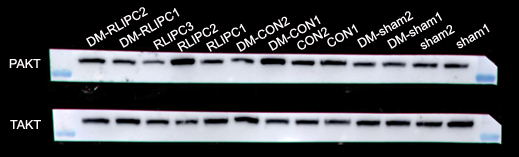


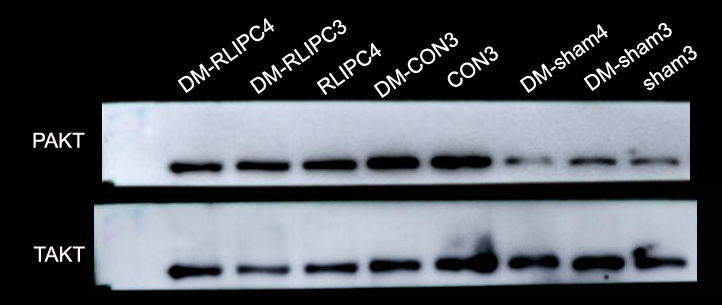


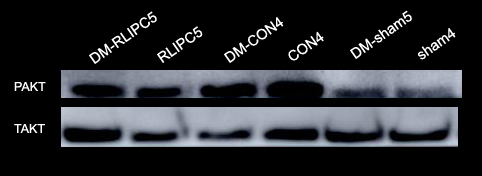


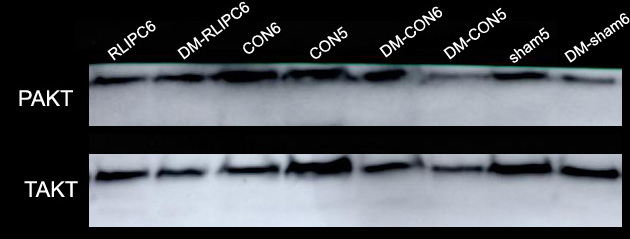


Figure 6D


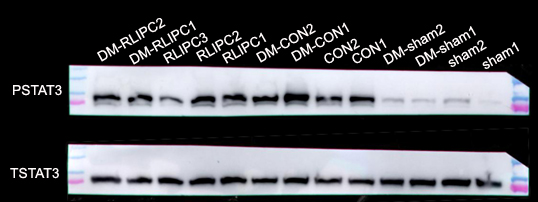


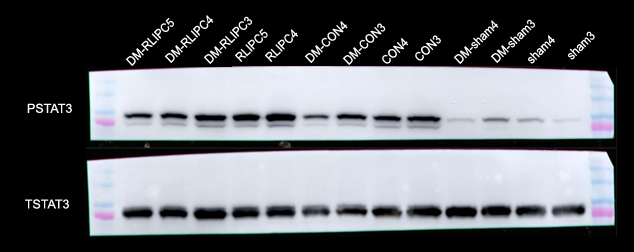


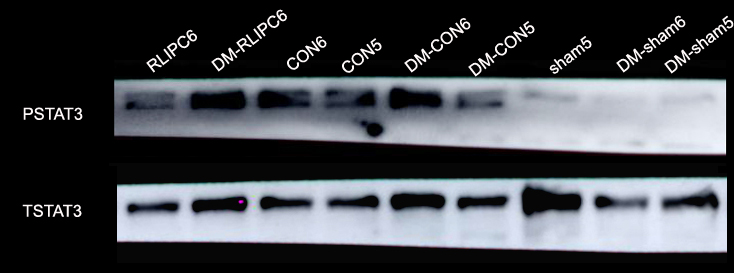


Figure 6E


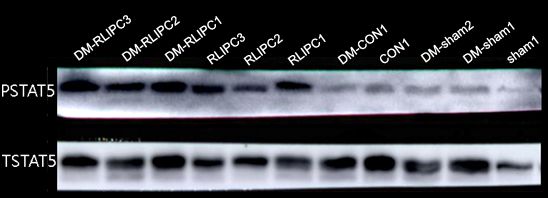


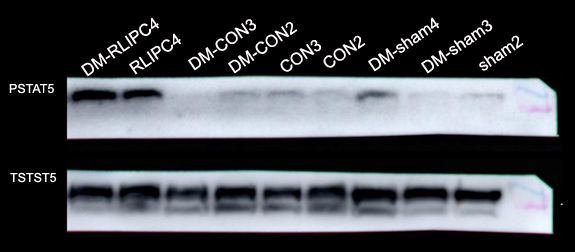


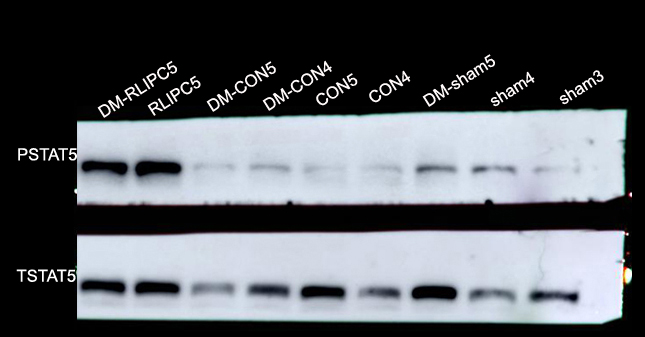


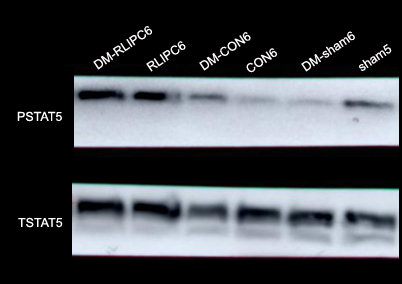

Supplement: Supplementary file 1 — Supplementary Information. [file 41598_2021_81422_MOESM1_ESM.docx]
